# Supplementary material for: One-pot synthesis of hyperbranched polymers via visible light regulated switchable catalysis
Source: Nat Commun. 2023 Mar 23;14:1622. doi: 10.1038/s41467-023-37334-x (PMC10036521; doi:10.1038/s41467-023-37334-x)
Supplement: Supplementary file 1 — Supplementary Information [file 41467_2023_37334_MOESM1_ESM.pdf]

Supplementary Information

One-pot synthesis of hyperbranched polymers via visible light regulated switchable catalysis

Shuaishuai Zhu[1†], Maoji Zhao[1†], Hongru Zhou[1], Yingfeng Wen[1], Yong Wang[1*], Yonggui Liao[1], Xingping Zhou[1], Xiaolin Xie[1]

[1]School of Chemistry and Chemical Engineering, Huazhong University of Science and Technology, Wuhan 430074, China

*Corresponding to: tcwy@mail.hust.edu.cn (Y. W.)

†These authors contributed equally to this work: Shuaishuai Zhu, Maoji Zhao.

Table of Contents

Supplementary Note 1--- Materials

Supplementary Note 2---Characterization methods

Supplementary Note 3---Experimental procedures

Supplementary Note 4---CO-insertion study

Supplementary Note 5---Characterization of the hyperbranched polymers

Supplementary References

Supplementary Note 1---Materials

All manipulations involving air- and/or water-sensitive compounds were carried out using standard Schlenk techniques under dry argon. propylene oxide (PO), tetrahydrofuran (THF), and glycidyl acrylate (GA) were distilled from calcium hydride (CaH_2) under argon atmosphere. Methylene chloride (CH_2Cl_2) and toluene were distilled under argon atmosphere from sodium/benzophenone. Carbon monoxide (purity of 99.99%) were provided from Wuhan Huaerwen Gas Ltd. and used as received. Diglycidyl ether of bisphenol F (DGEBF, YD-F170) was supported by KUNDO Chemical Co., Ltd. 2-ethyl-4-methylimidazole (EMI-2,4) used as cured agent was purchased from Aladdin Industrial Corporation. Other reagents and chemicals were used without further purification.

Supplementary Note 2---Characterization methods

NMR: ^{1}H NMR and ^{13}C NMR spectra were recorded on a Varian INOVA-400 MHz type (^{1}H, 400 MHz and ^{13}C, 100 MHz) spectrometer. Chemical shifts are reported in ppm from the internal standard, tetramethylsilane (0 ppm) for ^{1}H. Data are presented as follows: chemical shift, multiplicity (s = singlet, d = doublet, m = multiplet and/or multiplet resonances, br = broad), coupling constant in hertz (Hz), and signal area integration in natural numbers.

SEC: Size exclusion chromatography (SEC) was performed at 35 ^{o}C using polystyrene standards for calibration on Waters 410 GPC instrument with THF as eluent, using differential detector, multi-angle laser light scattering (MALLS) detector (Wyatt Technology, DAWN EOS), and viscosity detector.

ESI-MS: ESI-MS analytical measurements were performed in dichloromethane solutions on a SolariX 7.0T spectrometer (Bruker Daltonics).

DSC**:** The thermogram were measured using DSC Q20 (DuPont TA Instruments). A sealed empty crucible was used as a reference, and the DSC was calibrated using indium. Samples were heated from room temperature to 190 °C, at a rate of 10 $°C·min^{-1}$, under helium flow, and were kept at 190 °C for 2 mins to erase the thermal history. Subsequently, the samples were cooled to -10 °C, at a rate of 10 $°C·min^{-1}$, and kept at -10 °C for further 2 mins, followed by a heating procedure from -10 °C to 190 °C, at a rate of 10 $°C·min^{-1}$.

DMA: The dynamic mechanical properties were tested on a dynamic mechanical analyzer (DMA) 8000 (PerkinElmer Instruments, Inc., USA) at a frequency of 1 Hz. Rectangular samples with the dimension

of 40 × 10 × 3 mm^3 were measured using the three-point bending mode at a temperature range from 25 to 200 °C.

Pendulum impact testing: The test of impact strength was conducted through the plastic pendulum impact testing machine PTM7000 (Shenzhen SUNS Technology Stock Co., LTD., China).

Supplementary Note 3---Experimental procedures

Synthesis of (salen)Co^{III}Cl[1]

Salen ligand. To a solution of (R, R)-1,2-cyclohexanediamine (0.57 g, 5 mmol) in ethanol (40 mL) in a round-bottomed flask, 3,5-di-tert-butyl-2-hydroxybenzaldehyde (2.34 g, 10 mmol) in ethanol (50 mL) was slowly added. The mixture was heated to reflux for 6 hours at 80 °C and then cooled to room temperature. After filtration and dried in vacuum, a yellow solid was obtained with 95% yield. ^{1}H NMR ($CDCl_3$, 400 MHz): δ 1.26 (s, 18H), 1.45 (s, 20H), 1.64-2.0 (m, 6H), 3.37(s, 2H), 7.03 (s, 2H), 7.34 (s, 2H), 8.35 (s, 2H), 13.72 (s, 2H).

(Salen)Co^{II}. To a solution of salen ligand (4.37 g, 8 mmol) in CH_2Cl_2 (50 mL), anhydrous cobalt acetate (1.77 g, 10 mmol) in ethanol (25 mL) was slowly added under Ar. The reaction mixture was allowed to stirred for 30 min at room temperature and another 15 min at 0 °C, then, it was filtrated and washed with cold methanol. A red solid was collected and dried in vacuum at 40 °C with 96% yield.

(Salen)Co^{III}Cl. Recrystallized (salen)Co^{II} (6.0 g, 9.9 mmol) and *p*-toluenesulfonic acid monohydrate (2.0 g, 10.5 mmol, 1.06 equiv) were added to a 250 mL round-bottomed flask charged with a Teflon stir bar. Dichloromethane (100 mL) was added to the reaction mixture and stirred vigorously for a minimum of 30 min (open to the atmosphere at room temperature). The solvent was removed by rotary evaporation and the solid was further dried under reduced pressure. The resulting solid was suspended in pentane and filtered to afford green (salen)Co–OTs as the monohydrate with 99% yield. Additional characterization: ^{1}H NMR (DMSO-d_6, 400 MHz) d 1.30 (s, 18H), 1.55–1.61 (m, 2H), 1.74 (s, 18H), 1.87–1.93 (m, 2H), 1.98–2.01 (m, 2H), 2.27 (s, 3H), 3.04–3.08 (m, 2H), 3.58–3.62 (m, 2H), 7.09 (d, *J*=8.0 Hz, 2H), 7.43–7.47 (m, 6H), 7.81 (s, 2H).

Methylene chloride (200 mL) was added to a 500 mL separatory funnel. Tosylate catalyst (5.0 g) was added to the funnel and agitated until the solid completely dissolved. The organic layer was rinsed with saturated aqueous NaCl (3 x 200 mL). The organic layer was dried over Na_2SO_4 and concentrated under reduced pressure. The solid was suspended in pentane and filtered to afford very dark green powder with

85% yield. Additional characterization: ^{1}H NMR (DMSO-d_6, 400 MHz): δ 1.28 (s, 18H), 1.52-1.62 (m, 2H), 1.72 (s, 18H), 1.85-1.97(m, 2H), 1.97-2.05 (m, 2H), 3.04-3.11 (m, 2H, CyH), 3.58-3.66 (m, 2H), 7.45 (d, 4H), 7.78 (s, 2H).

Synthesis of compound 2a via (salen)Co^{III}Cl catalyzed ring-opening of GA under the CO atmosphere

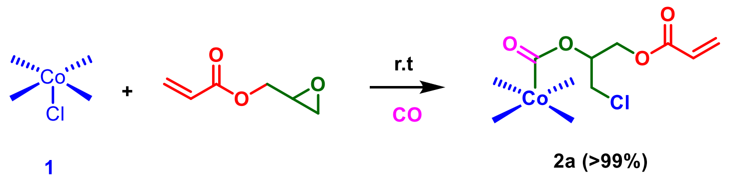

In an argon filled glove box, (salen)Co^{III}Cl complex (63.9 mg, 0.1 mmol), GA (23 µL, 0.2 mmol), and THF (1 mL) were charged into a 10 mL ampoule, which was wrapped in aluminum foil and equipped with a magnetic stir bar. The ampoule was then taken out of the glove box and allowed to be stirred under 1 atm CO at room temperature for 8 h. Dark green powder was obtained after GA and THF were evaporated under vacuum with 98% yield.

OMR-SCVP of GA with 2a as the inimer

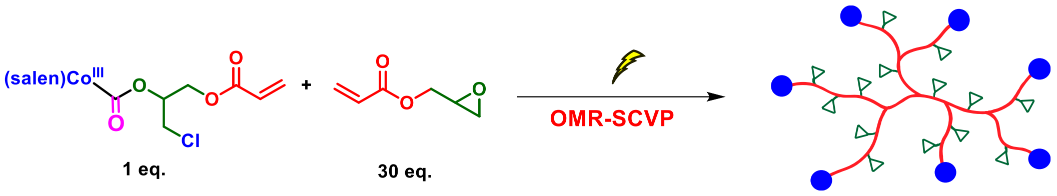

See the Methods section of the main manuscript for detailed experimental procedures.

Visible light regulated switchable catalysis from ring-opening to OMR-SCVP with GA as a heterofunctional monomer.

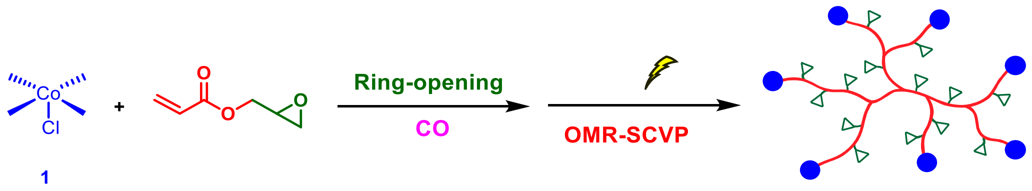

See the Methods section of the main manuscript for detailed experimental procedures.

Chain extension of *hb*-PGA-Co^{III}(salen) with MA

In an argon filled glove box, *hb*-PGA-Co^{III}(salen) (0.96 g, $M_{n,\ SEC}$ = 9.6 kDa, 0.1 mmol), MA (2.70 mL, 30 mmol), and THF (2 mL) were charged into a 25 mL ampoule, which was wrapped in aluminum foil and equipped with a magnetic stir bar. The ampoule was then taken out of the glove box and allowed to stirred at 25 ºC under irradiation of a household white LED lamp with light intensity of 10 mW·cm^{-2}. A

small aliquot of the polymerization mixture was taken out for ^1H NMR spectroscopy and the remained crude mixture was precipitated in cold methanol.

Preparation of *hb*-PGA-TEMPO/DGEBF

A certain amount of *hb*-PGA-TEMPO and DGEBF were dissolved in methylene chloride. Then most of solvent was rapidly removed using a rotary evaporator. The obtained mixture was further dried in a vacuum at 40 °C for 6 h. After which, 6 wt% EMI-2, 4 relative to epoxy resin was incorporated into the mixture and degassed for several minutes. Finally, the uncured mixture was decanted into a PTFE mold and underwent a programmed curing of 60 °C for 2 h, 150 °C for 8 h. For comparison, neat DGEBF were fabricated using the same process.

Synthesis of alkoxycarbonyl cobalt complex 3[2]

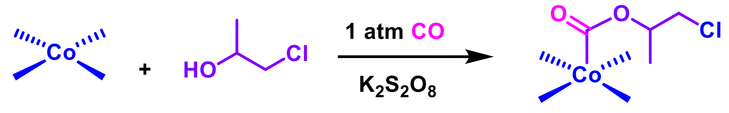

A mixture of (salen)Co^{II} (3 g, 4.95 mmol) and excess potassium persulfate was added to CH_2Cl_2. After degassed with CO (balloon) for 10 s, the reaction mixture was then treated with the respective alcohol (2.33 g, 24.75 mmol) and stirred for the indicated time in the dark, at room temperature, under 1 atm of CO. Reactions were tracked by TLC (3% MeOH in CH_2Cl_2). Upon completion, the product was precipitated by addition of heptane, filtered through Celite and washed several times with heptane to obtain a dark green powder, yield 91%.

Procedure for the synthesis of linear PGA

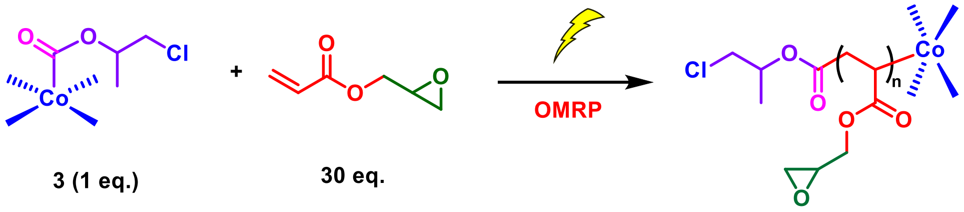

In an argon filled glove box, complex **3** (72.4 mg, 0.1 mmol), GA (0.34 mL, 3 mmol), and THF (1.16 mL) were charged into a 10 mL ampoule equipped with a magnetic stir bar. The ampoule was then taken out of the glove box and allowed to be stirred at 25 °C under irradiation of a household white LED lamp with light intensity of 10 mW·cm^{-2}. A small aliquot of the polymerization mixture was taken out for ^1H NMR spectroscopy and the remained crude mixture was precipitated in cold methanol.

Supplementary Note 4---CO-insertion study

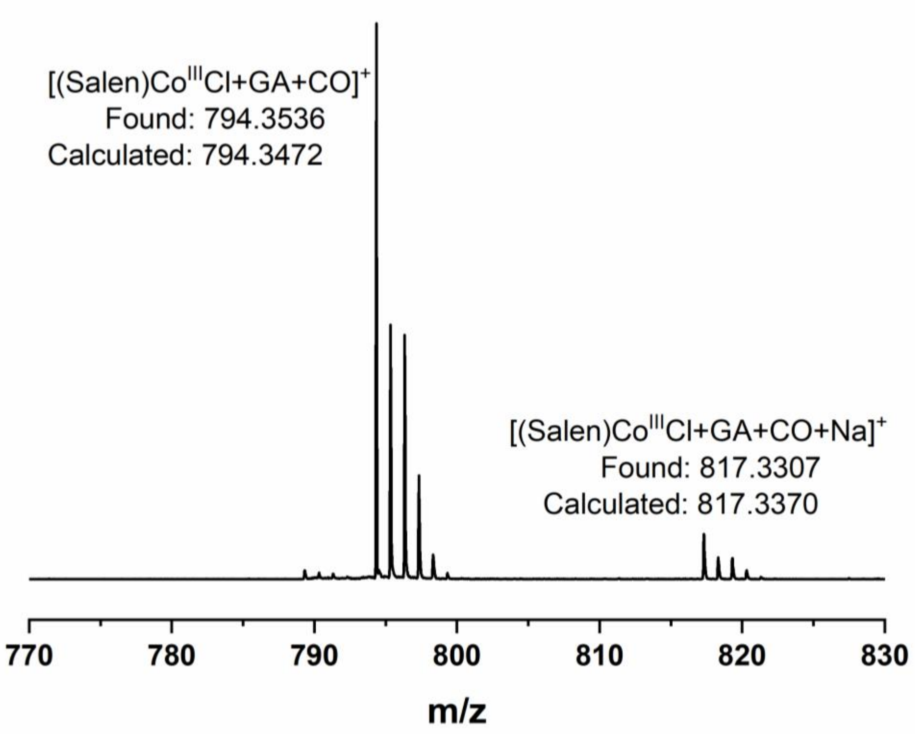

Supplementary Figure 1. ESI-MS spectrum of compound **2a**.

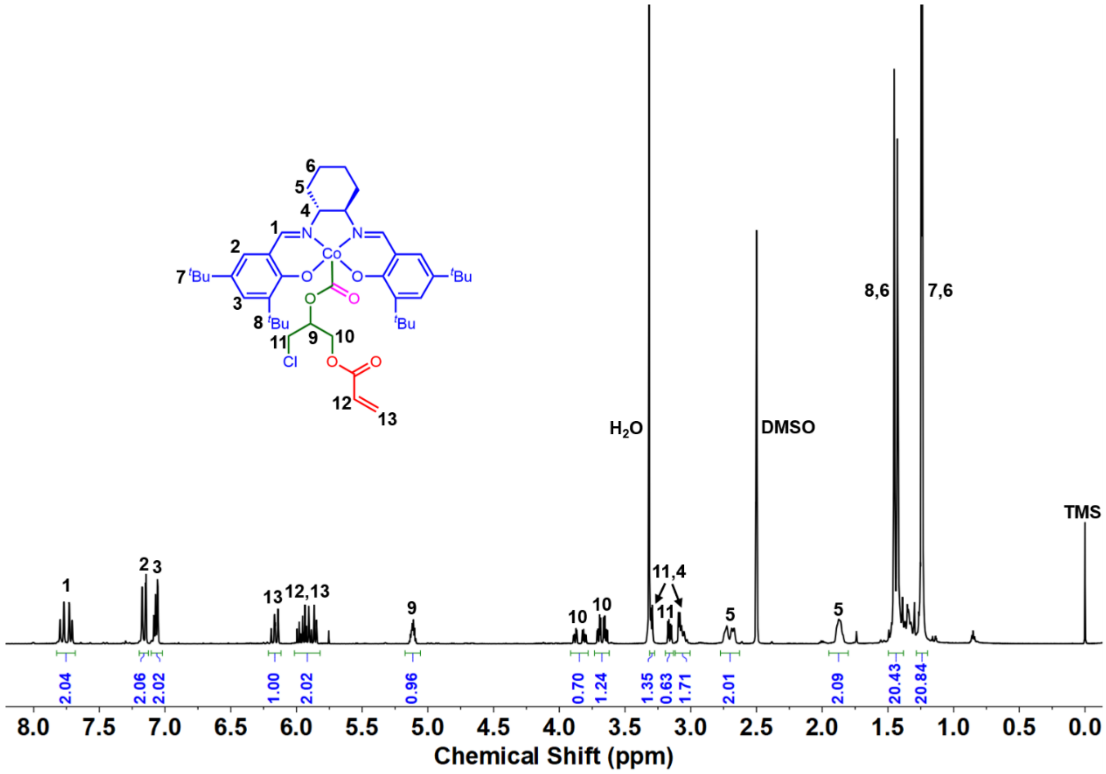

Supplementary Figure 2. ^{1}H NMR spectrum of compound **2a** in DMSO-d_6 at 25 °C. ^{1}H NMR (400 MHz, DMSO-d_6) δ 7.83 – 7.69 (m, 2H), 7.22 – 7.13 (m, 2H), 7.12 – 7.03 (m, 2H), 6.17 (ddd, J = 17.2, 14.1, 1.6 Hz, 1H), 6.02 – 5.83 (m, 2H), 5.15 – 5.08 (m, 1H), 3.92 – 3.62 (m, 2H), 3.31 – 3.01 (m, 4H), 2.81 – 2.64 (m, 2H), 1.87 (s, 2H), 1.44 (dd, J = 15.7, 2.5 Hz, 20H), 1.24 (t, J = 2.8 Hz, 20H).

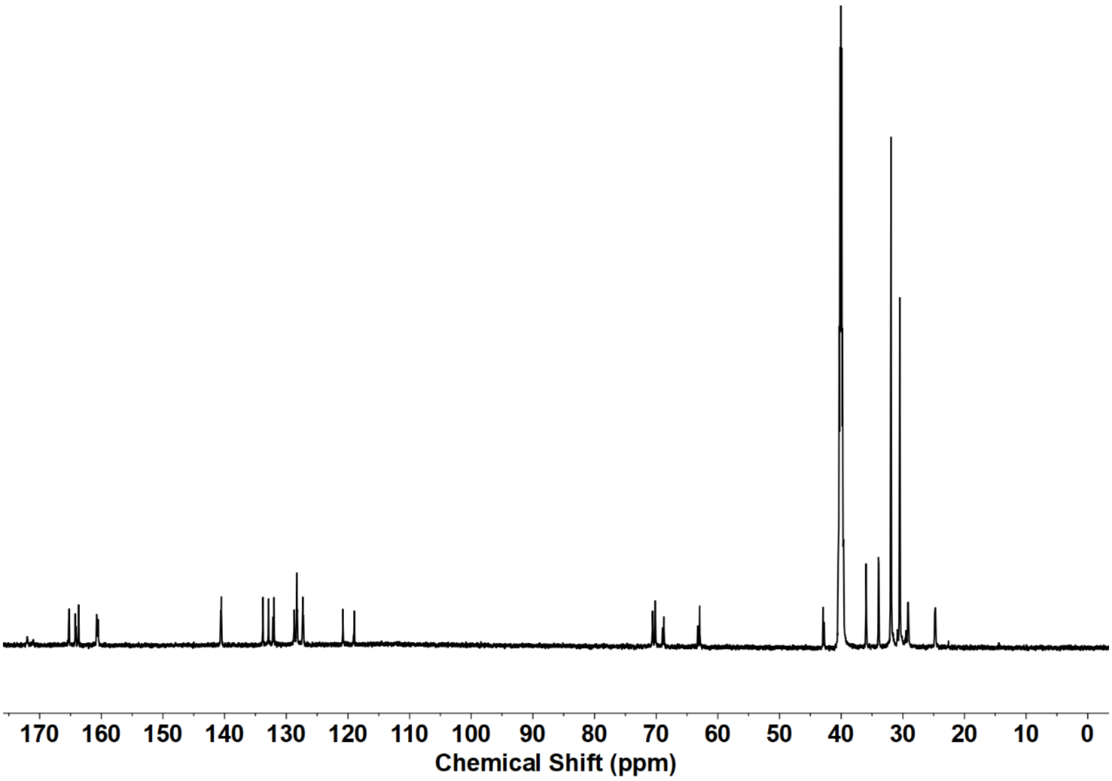

Supplementary Figure 3. ^{13}C NMR spectrum of compound **2a** in DMSO-d_6 at 25 °C. ^{13}C NMR (100 MHz, DMSO-d_6) δ 172.03, 165.21, 164.23, 163.68, 160.75, 160.51, 140.63, 140.53, 133.78, 132.88, 132.18, 132.00, 128.72, 128.33, 128.27, 127.32, 127.24, 120.82, 119.06, 118.97, 70.58, 70.17, 68.76, 63.25, 62.94, 42.93, 42.73, 35.96, 35.91, 33.96, 33.88, 31.92, 30.49, 29.18, 29.13, 29.08, 24.83, 24.77, 24.70.

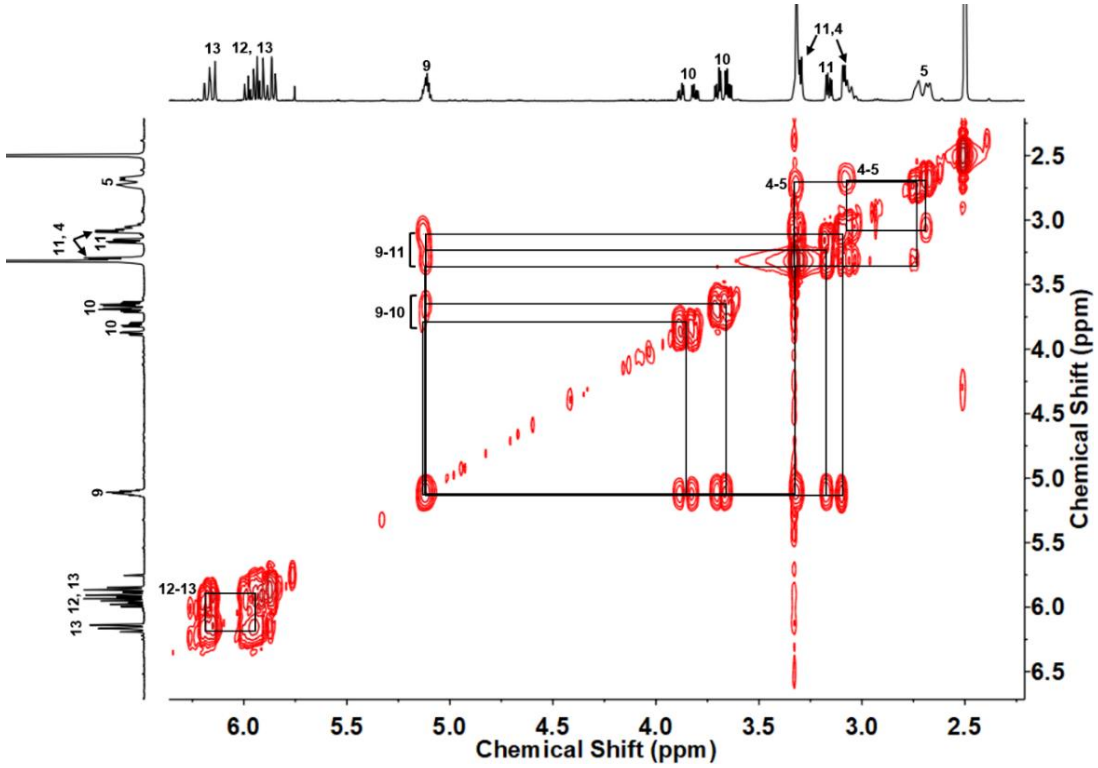

Supplementary Figure 4. COSY NMR spectrum of compound **2a** in DMSO-d_6 at 25 °C.

Supplementary Note 5---Characterization of the hyperbranched polymers

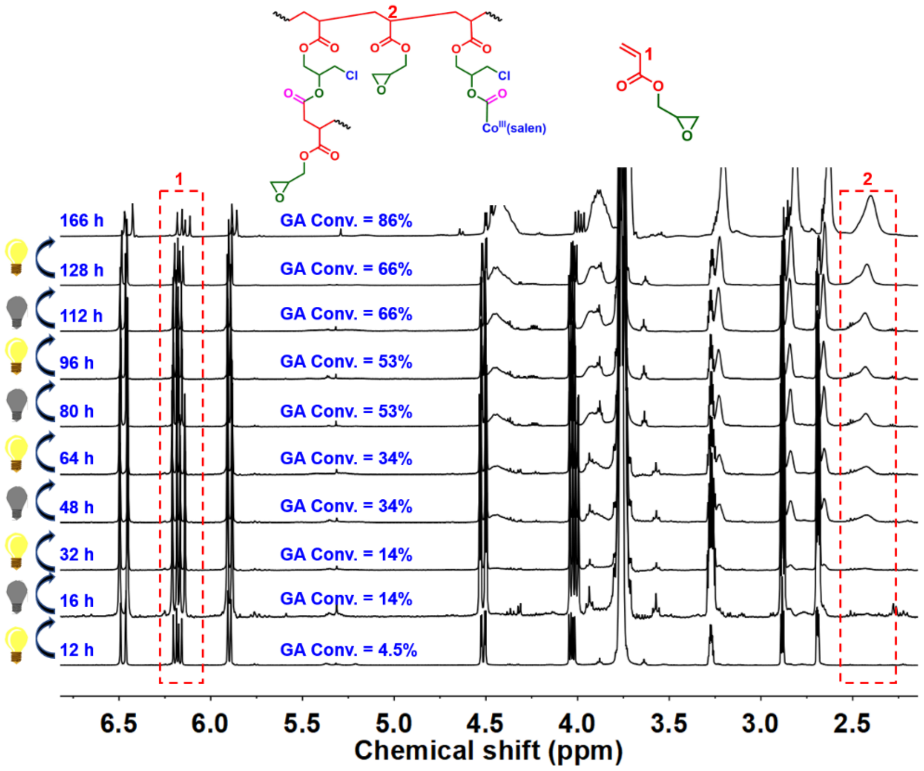

Supplementary Figure 5. Evolution of ^{1}H NMR spectra for the "ON/OFF" switching of OMR-SCVCP stage by light ([GA]/[**1**] = 30/1, [GA] = 2 M in THF).

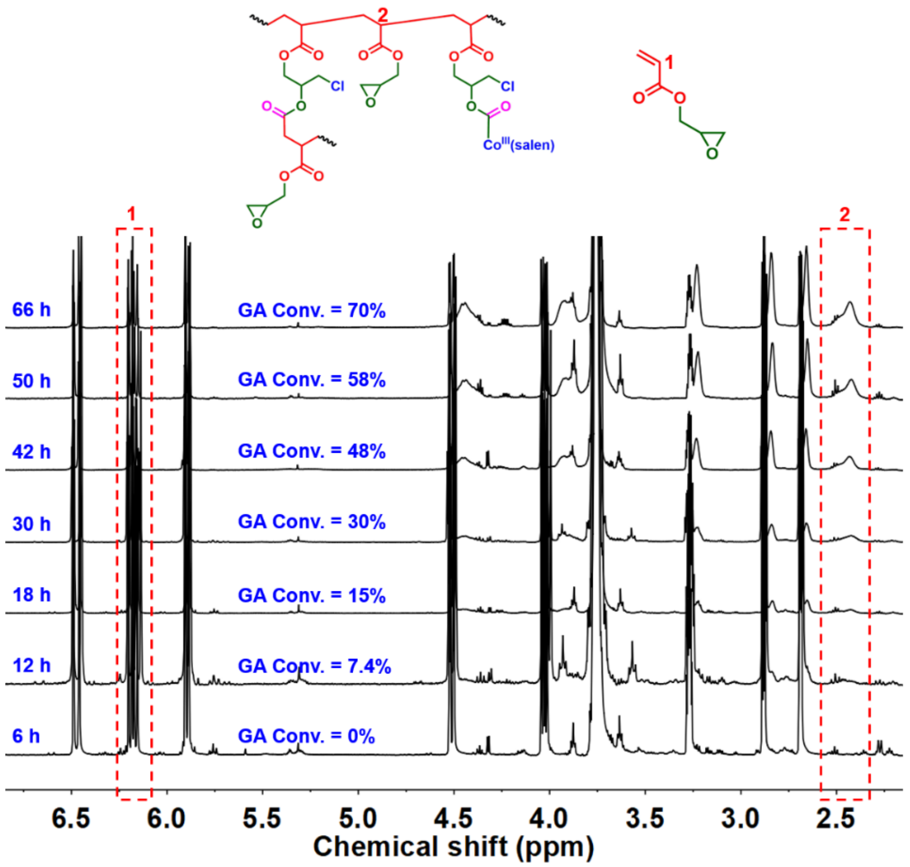

Supplementary Figure 6. Evolution of ^{1}H NMR spectra for the OMR-SCVCP stage ([GA]/[**1**] = 30/1, [GA] = 2 M in THF).

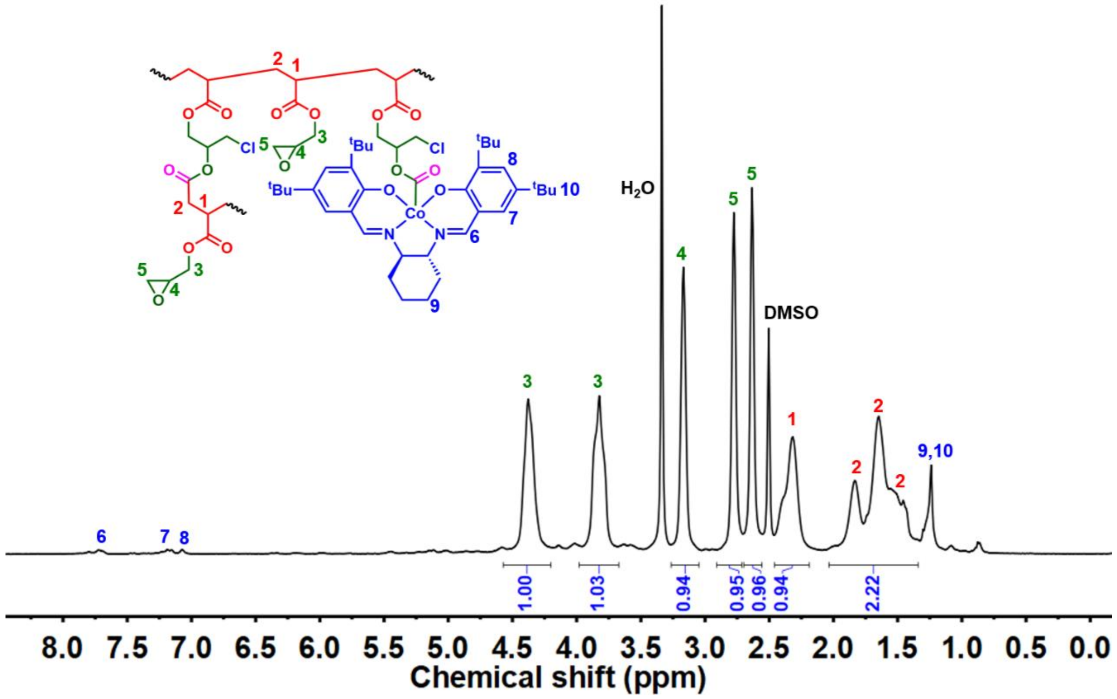

Supplementary Figure 7. ^{1}H NMR spectrum of *hb*-PGA-Co^{III}(salen) in DMSO-d_6 at 25 °C (Table 1, entry 3).

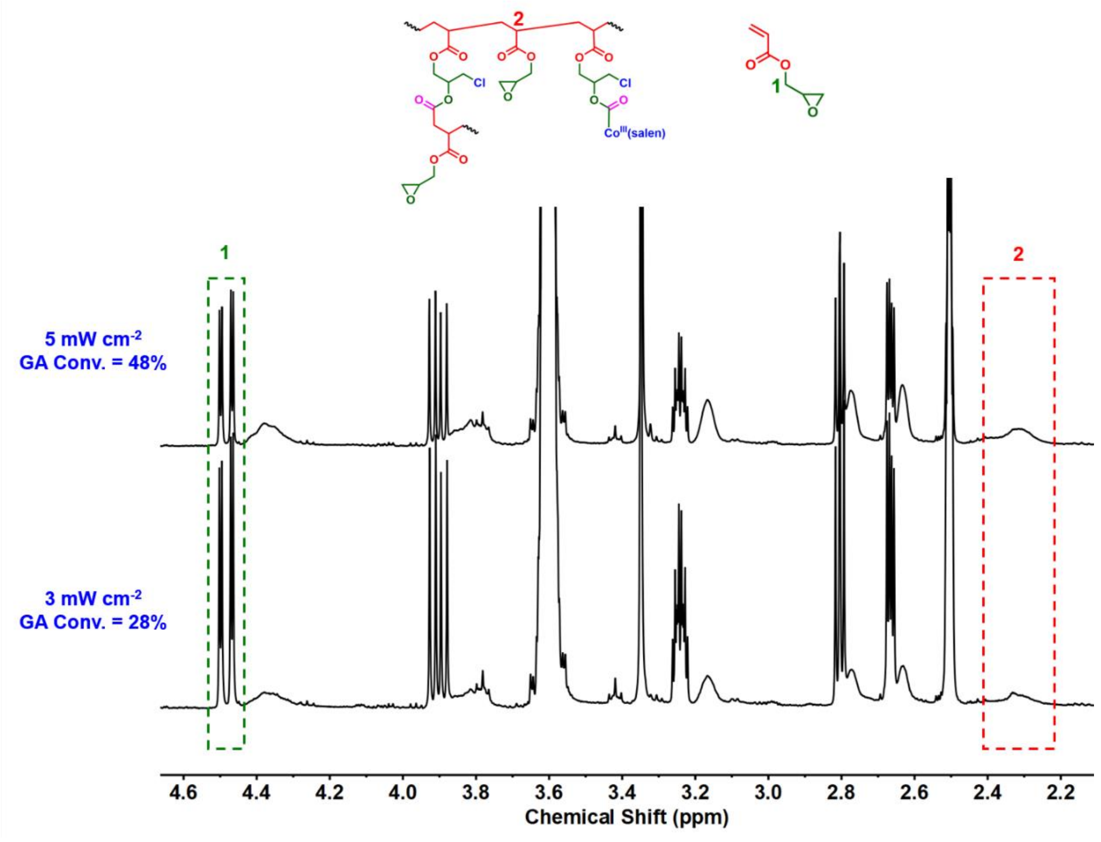

Supplementary Figure 8. ^{1}H NMR spectra of reaction mixtures of cobalt-mediated switchable catalytic transformation of GA under the intensities of 3 and 5 mW·cm^{-2}, respectively([GA]/[**1**]=30/1, 65 h, [GA] = 2 M in THF).

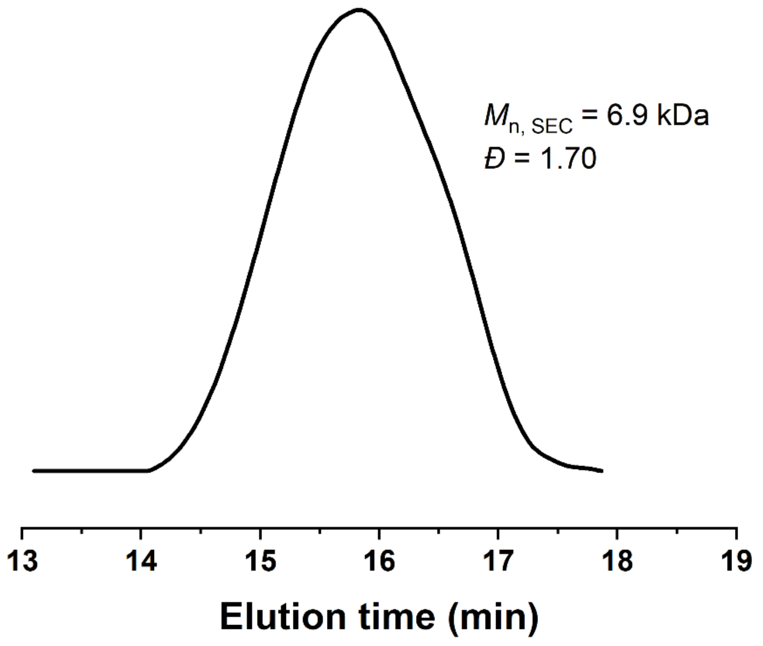

Supplementary Figure 9. SEC trace of *hb*-PGA-Co^{III}(salen) (Table 1, entry 4).

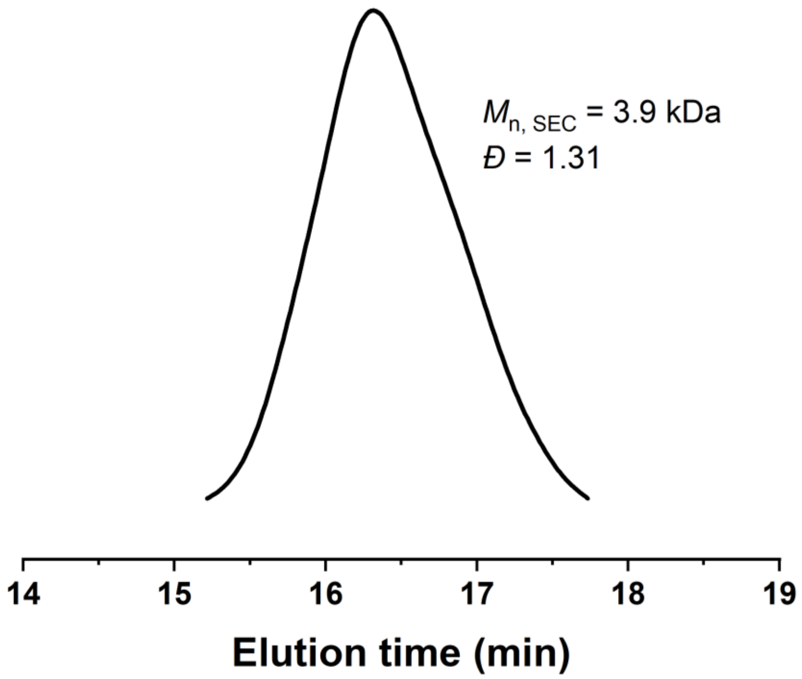

Supplementary Figure 10. SEC trace of *hb*-PGA-Co^{III}(salen) (Table 1, entry 5).

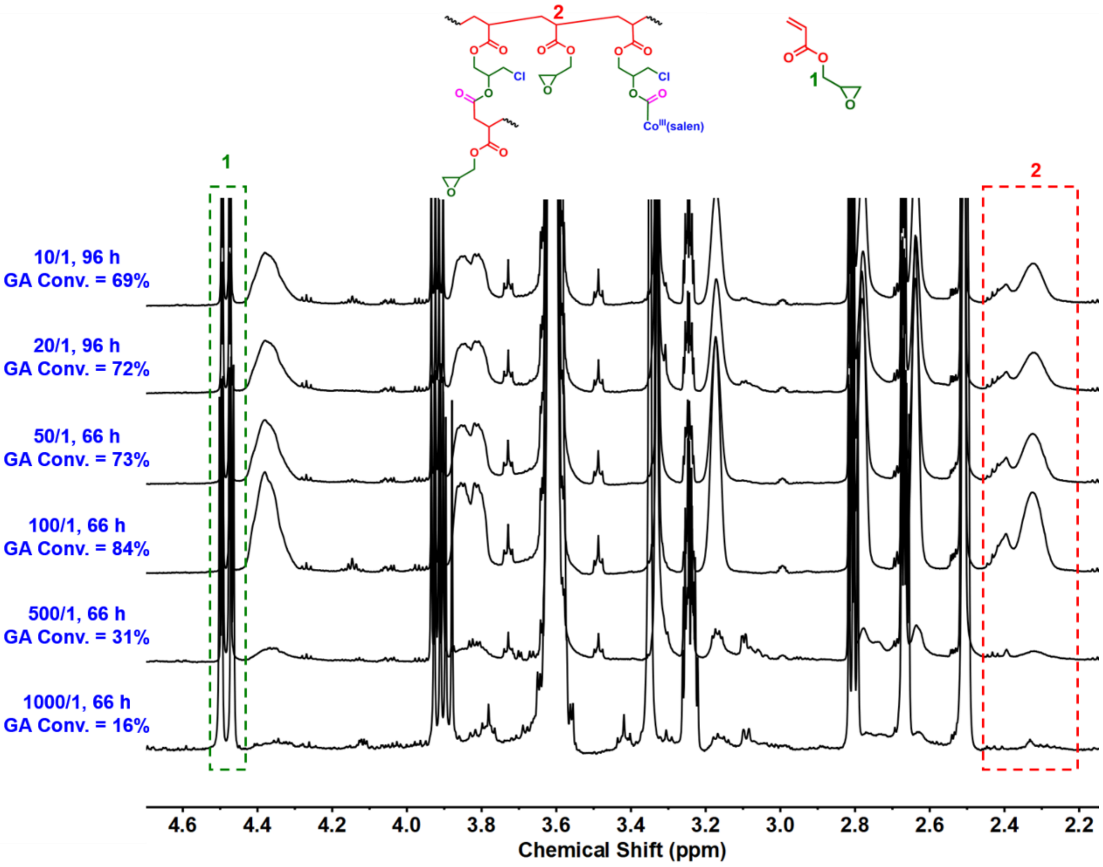

Supplementary Figure 11. ^{1}H NMR spectra of reaction mixtures of cobalt-mediated switchable catalytic transformation of GA under the [GA]/[**1**] feed ratio of 10/1, 20/1, 50/1, 100/1, 500/1 and 1000/1 respectively(10 mW·cm^{-2}, [GA] = 2 M in THF).

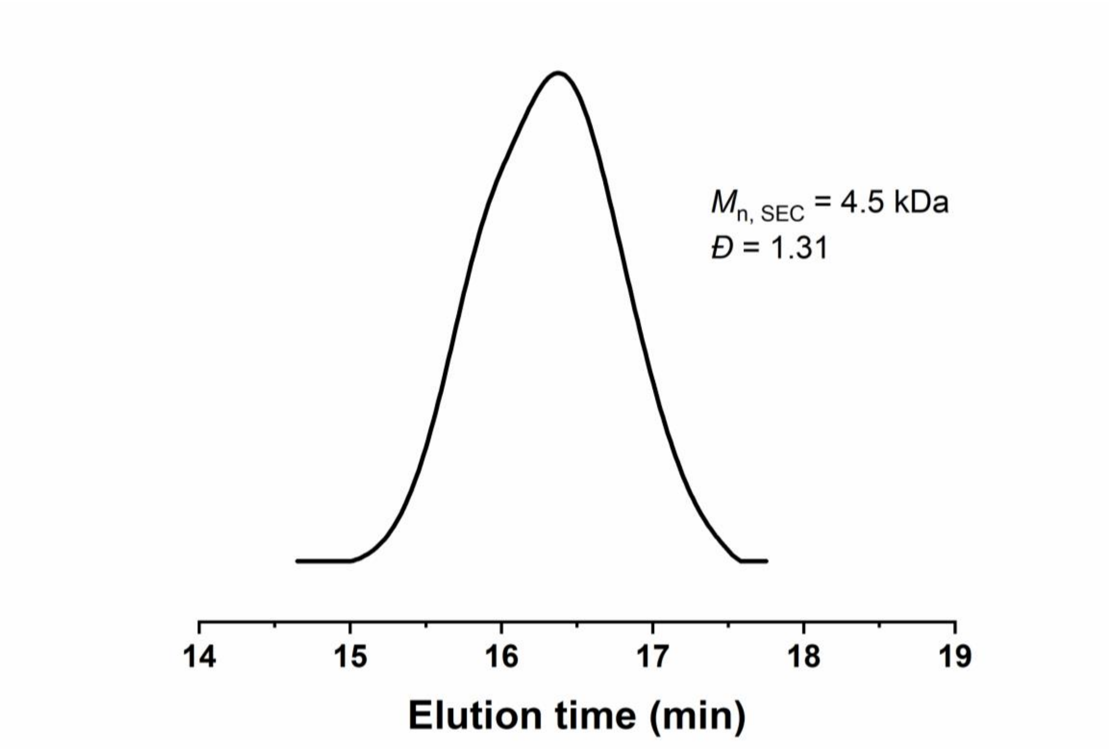

Supplementary Figure 12. SEC trace of *hb*-PGA-Co^{III}(salen) (Table 1, entry 7).

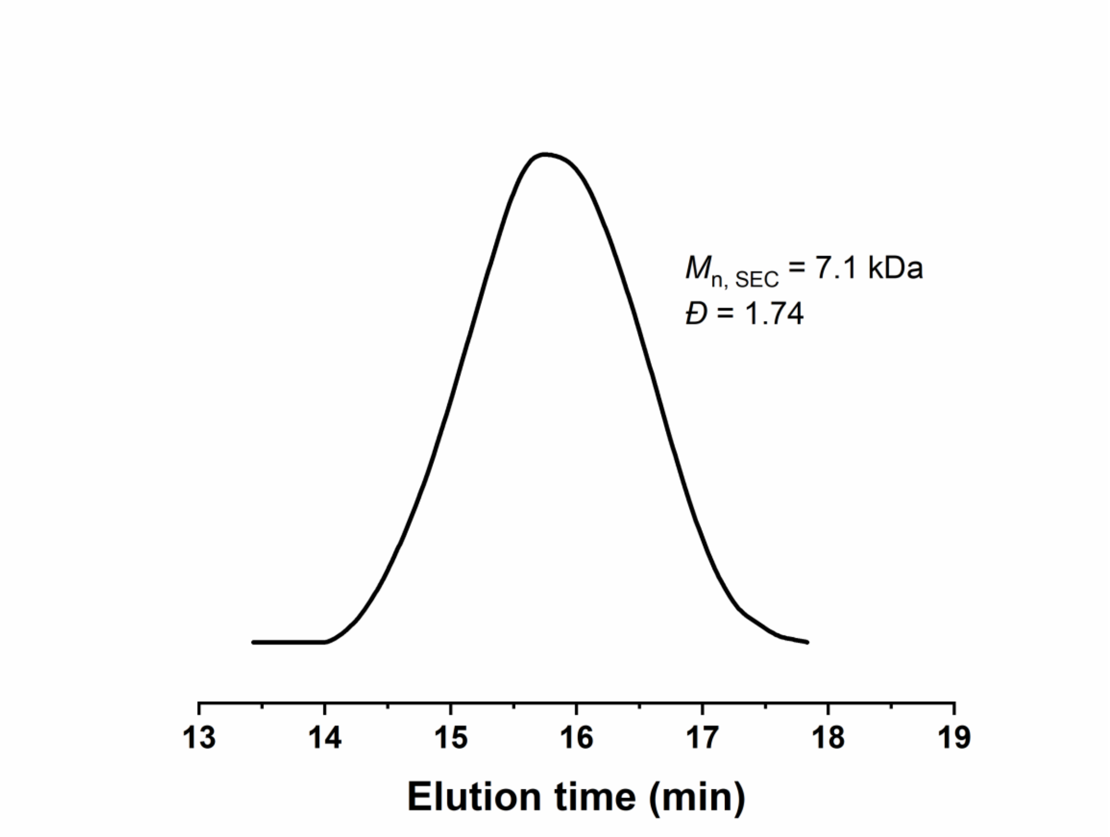

Supplementary Figure 13. SEC trace of *hb*-PGA-Co^{III}(salen) (Table 1, entry 8).

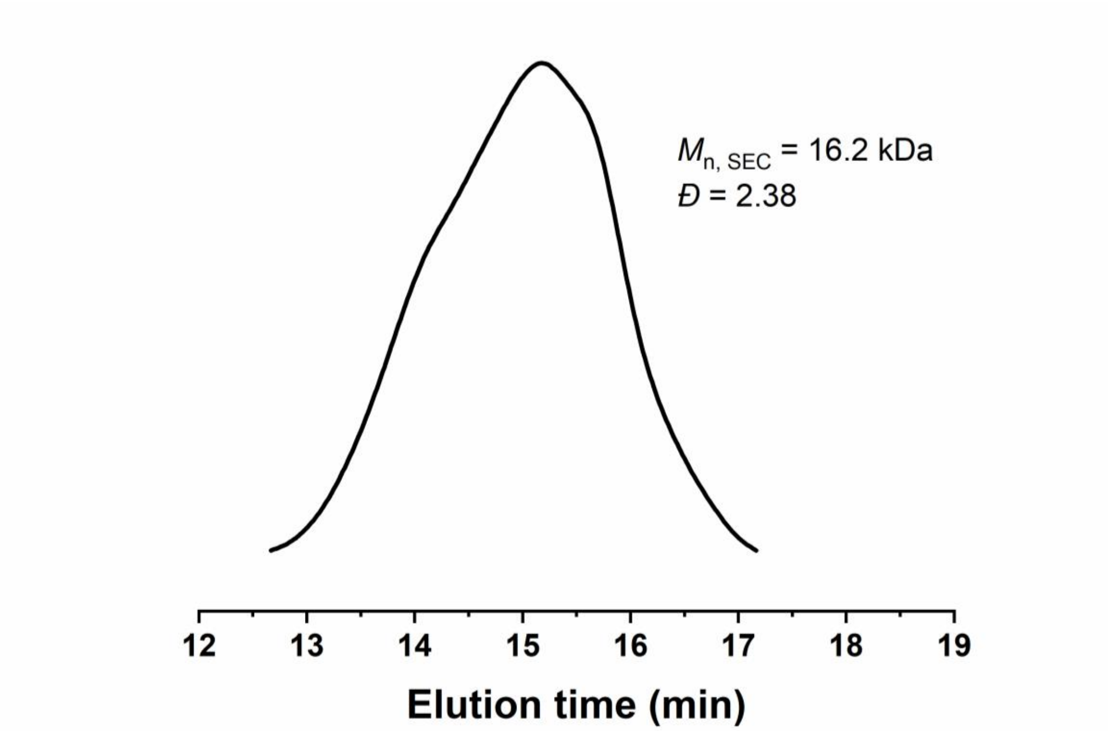

Supplementary Figure 14. SEC trace of *hb*-PGA-Co^{III}(salen) (Table 1, entry 9).

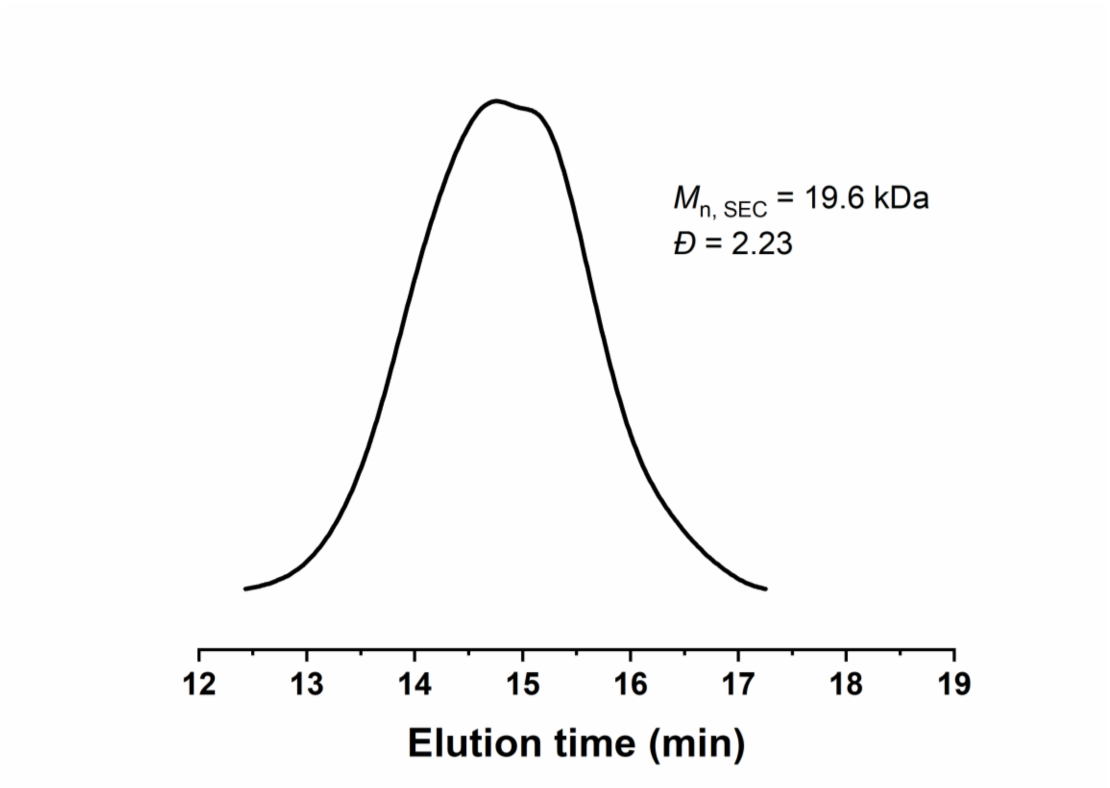

Supplementary Figure 15. SEC trace of *hb*-PGA-Co^{III}(salen) (Table 1, entry 10).

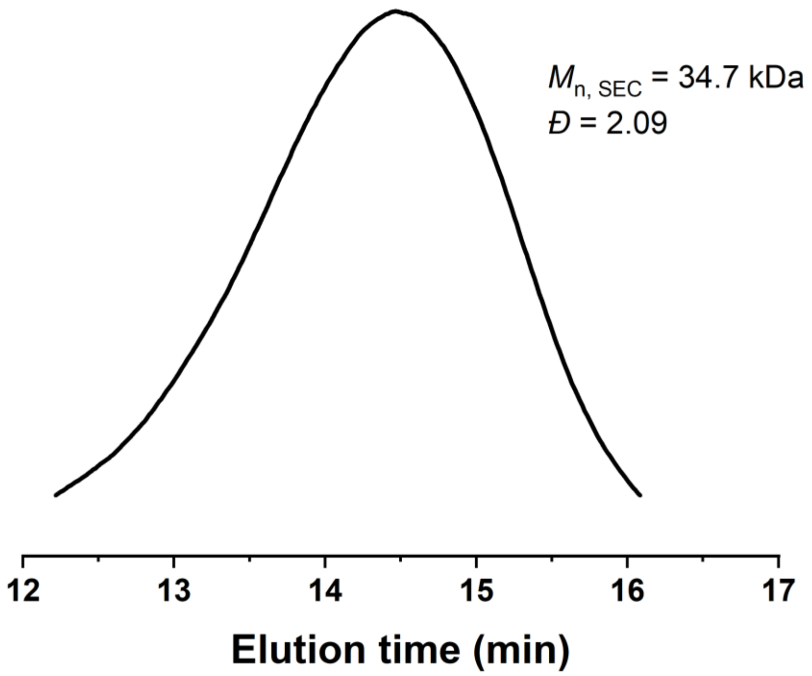

Supplementary Figure 16. SEC trace of *hb*-PGA-Co^{III}(salen) (Table 1, entry 11).

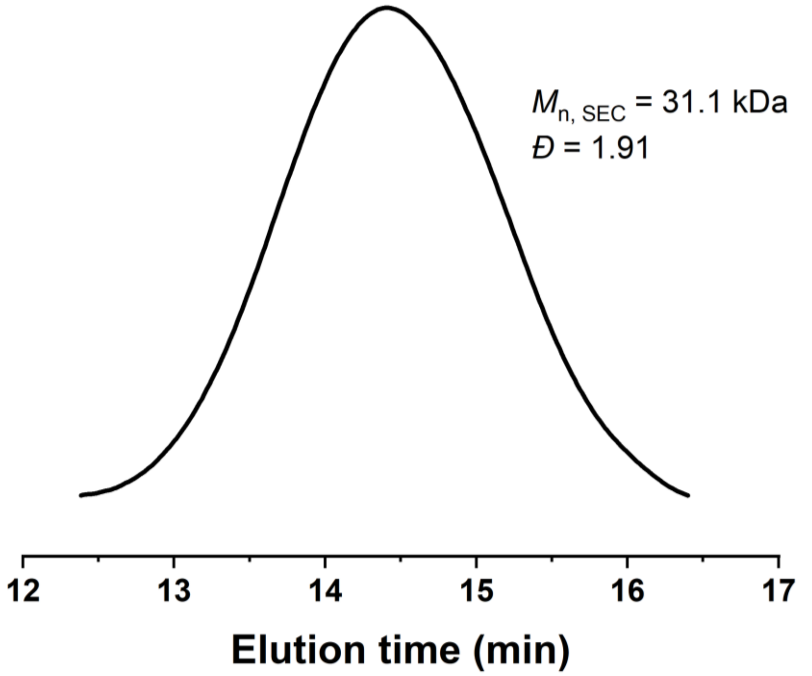

Supplementary Figure 17. SEC trace of *hb*-PGA-Co^{III}(salen) (Table 1, entry 12).

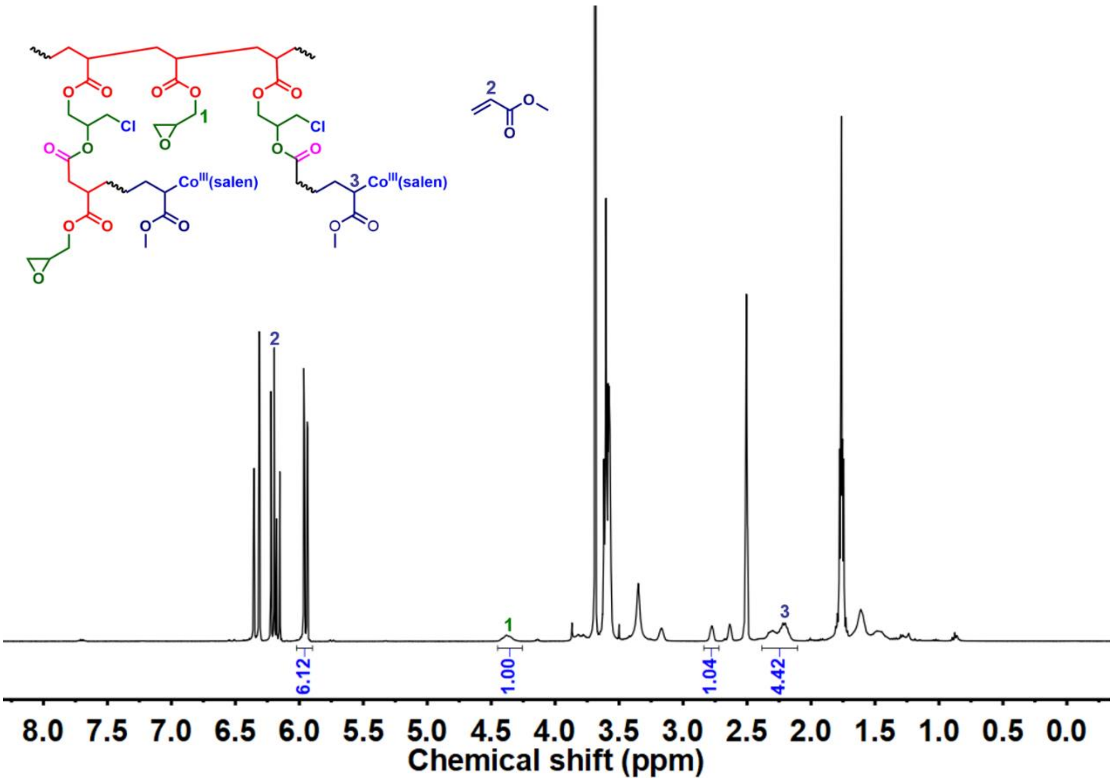

Supplementary Figure 18. ^{1}H NMR spectrum of reaction mixture of the chain extension reaction with MA ([*hb*-PGA-Co^{III}(salen)]/[MA]=1/300, performed in THF) in DMSO-d_6 at 25 ^{o}C.

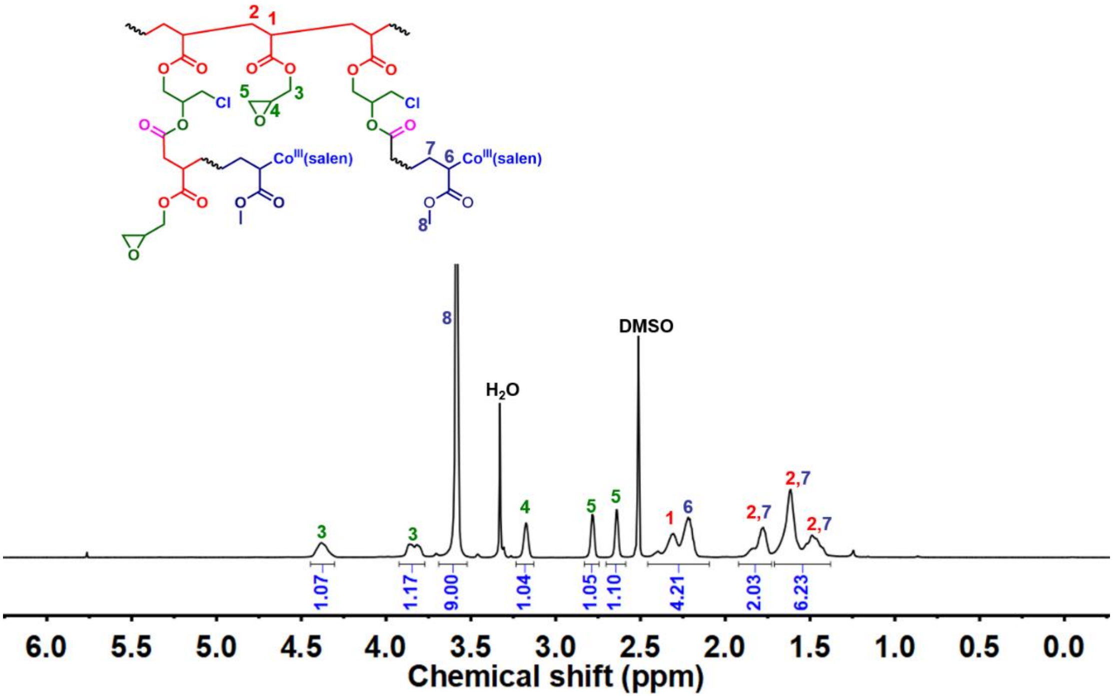

Supplementary Figure 19. ^{1}H NMR spectrum of *hb*-PGA-*g*-PMA-Co^{III}(salen) in DMSO-d_6 at 25 ^{o}C.

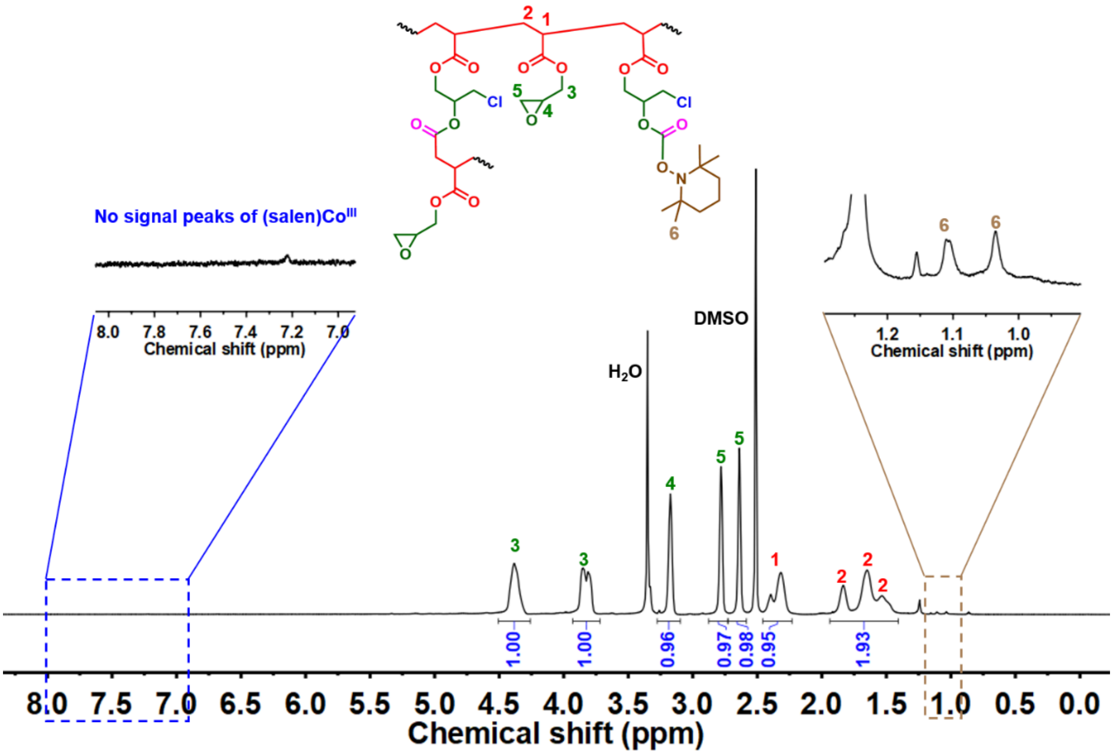

Supplementary Figure 20. ^{1}H NMR spectrum of *hb*-PGA-TEMPO in DMSO-d_6 at 25 °C ((Table 1, entry 6).

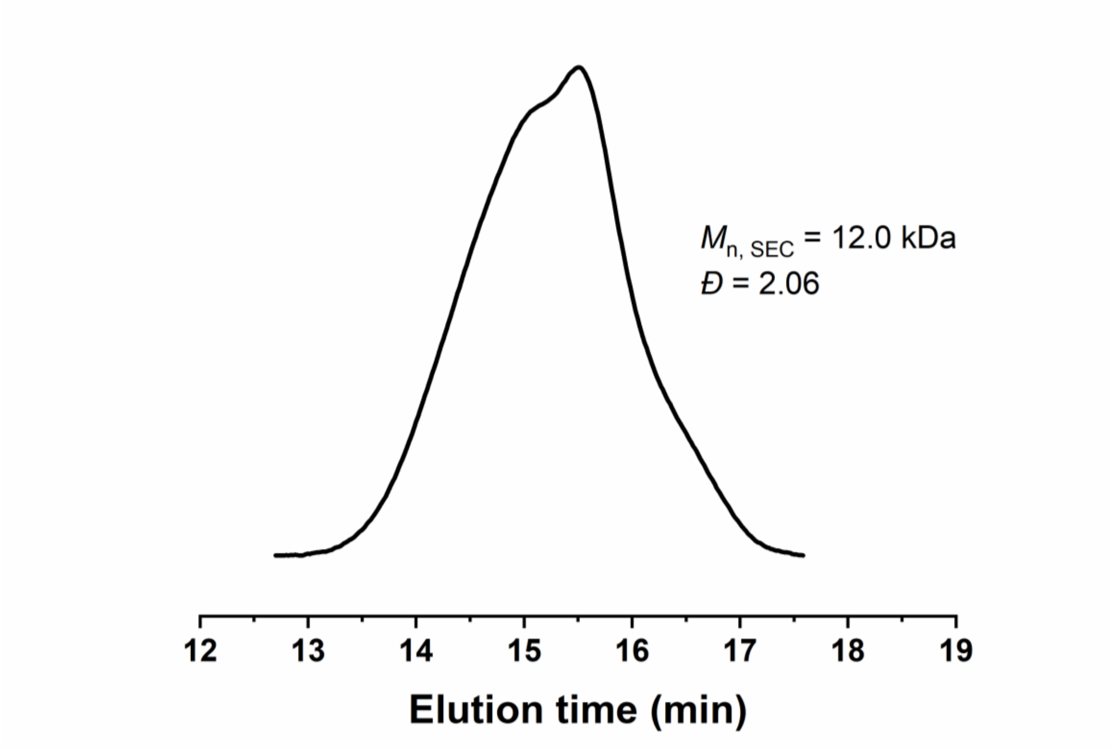

Supplementary Figure 21. SEC trace of *hb*-PGA-TEMPO (Table 1, entry 6).

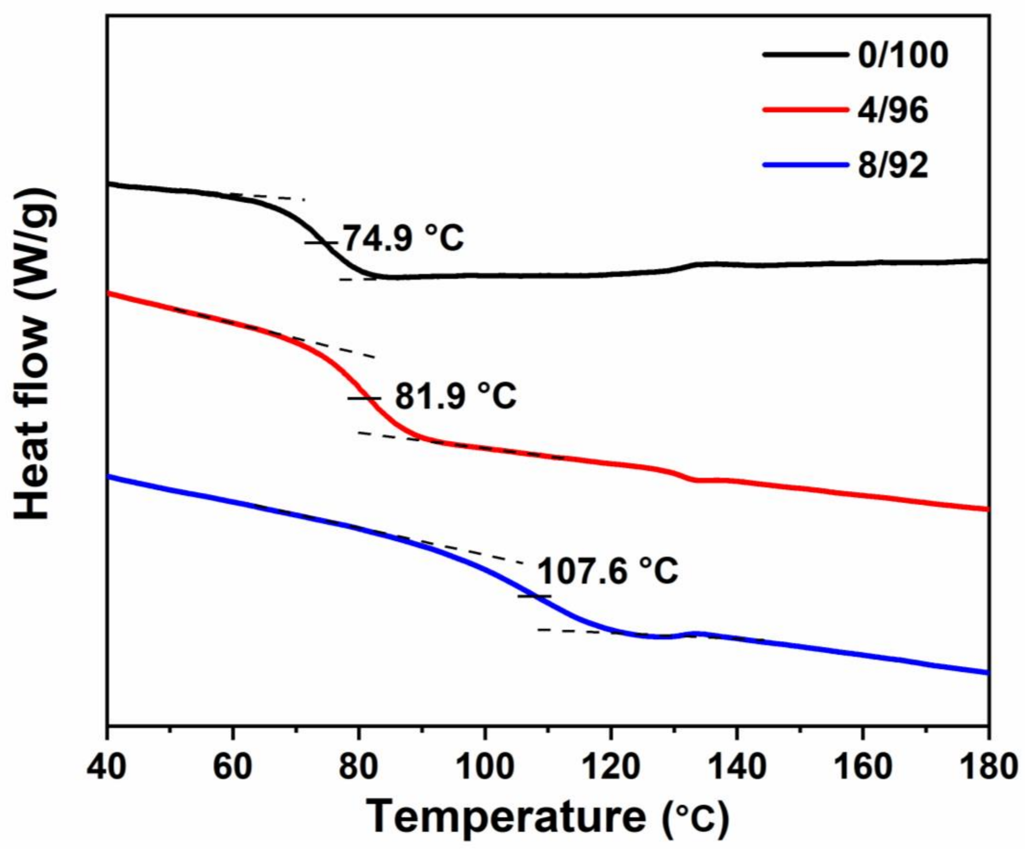

Supplementary Figure 22. The DSC results of the *hb*-PGA-TEMPO/DGEBF samples.

The cross-linking densities (v) of the *hb*-PGA-TEMPO/DGEBF samples were calculated from the following equation referring to rubber-like elasticity theory

$$v = \frac{E_R}{3RT}$$

where E_R is the storage modulus at rubbery state (T_g + 30 °C), T is the absolute temperature (T_g + 30 K), and R is the gas constant (8.314 J mol^{-1} K^{-1}). From the DMA results we could get the storage modulus and the absolute temperature.

Supplementary Table 1. Storage modulus and cross-linking density data for *hb*-PGA-TEMPO/DGEBF samples.

Sample	Modulus at 25 °C (MPa)	T_g (°C)	Modulus at T_g + 30 °C (MPa)	v (×10^3 mol/m^3)
0/100	6996	65.6	46	5.00
4/96	6468	87.3	78	8.01
8/92	6016	101.6	82	8.12

Supplementary References

1 Nielsen, L. P. C., Stevenson, C. P., Blackmond, D. G. & Jacobsen, E. N. Mechanistic investigation leads to a synthetic improvement in the hydrolytic kinetic resolution of terminal epoxides. *J. Am. Chem. Soc.* **126,** 1360-1362 (2004).

2 Wang, Y. et al. Switchable polymerization triggered by fast and quantitative insertion of carbon monoxide into cobalt-oxygen bonds. *Angew. Chem. Int. Ed.* **59**, 5988-5994 (2020).
